# Complete mitochondrail genome of *Corydoras panda* (Teleostei, Siluriformes, Callichthyidae, Corydoradinae)

**DOI:** 10.1080/23802359.2019.1660253

**Published:** 2019-09-03

**Authors:** Qiaolin Liu, Yi Liu, Tiaoyi Xiao, Baohong Xu

**Affiliations:** a Hunan Engineering Technology Research Center of Featured Aquatic Resources Utilization, Hunan Agricultural University, Changsha, PR China;; b Collaborative Innovation Center for Efficient, Health Production of Fisheries in Hunan Province, Changde, PR China

**Keywords:** *Corydoras panda*, mitochondrail genome, next-generation sequencing, phylogenetic analysis

## Abstract

We reported the complete mitochondrial genome yielded by next-generation sequencing of *Corydoras panda* in this study. The total length of the mitochondrial genome is 16,611 bp, with the base composition of 32.50% A, 26.30% T, 26.10% C, and 15.10% G, respectively. It contains two ribosomal RNA genes, 13 protein-coding genes, 22 transfer RNA genes, and a major non-coding control region (D-loop region). The arrangement of these genes is the same as that found in the Siluriformes. The complete mitogenomes of *C. panda* and other 18 species from Siluriformes were used for phylogenetic analysis using Neighbor-Joining method. The topology demonstrated that all species belong to nine genera are divided into three groups (Siluridae, Loricariidae, and Callichthyidae), and the *C. panda* was clustered with other species from genus *Corydoras*. *Corydoras panda* external morphological feature classification is consistent with the molecular classification results, so the information of the mitogenome could be used for future identification of *Corydoras* species.

*Corydoras panda*, belongs to Teleostei, Siluriformes, Callichthyidae, Corydoradinae, *Corydoras*, is usually named ‘panda mouse’ in Chinese ornamental fish market because of the external morphological feature of a light colored body with black blob covering their eyes (like the panda), a black spot the dorsal fin, and a spot on or near the base of the caudal fin.

We determined the mitochondrial genome sequence of the ‘panda mouse’ *C. panda* in order to distinguish and identify the species more accurately and carried out mitochondrial genome structure and phylogenetic analysis. The living body of ‘panda mouse’ was collected from the Red Star Ornamental Fish Market in Changsha, Hunan Province, China (113.03 E, 28.09 N). After anesthesia with MS-222 (3-Aminobenzoic acid ethyl ester methanesulfonate), dorsal muscle tissue was collected and preserved in 99% ethanol in Museum of Hunan Agricultural University. After DNA extraction (Tissue DNA Kit D3396-02, Omega, bio-tek) and sequencing library construction(Sangon Biotech, Shanghai), paired-end reads were sequenced using HiSeq XTen PE 150 of Illumina. BBduk and BLAST + were used to assess and monitor data quality. NOVOPlasty and SPAdes were used for de novo assembly. MITOS2 server and Geneious R11 (Liu et al. 2019; Tan et al. 2019) were used to predict and annotate the mitochondrial genome. Geneious Tree Builder was used for phylogenetic analysis and building phylogenetic tree.

Totally 23,481,486 high-quality clean reads (150 bp PE read length) were obtained. The total length of the *C. panda* mitochondrial genome is 16,611 bp (GenBank accession number: MN096581), with the base composition of 32.50% A, 26.30% T, 26.10% C, and 15.10% G, respectively. It contains two ribosomal RNA genes, 13 protein-coding genes, 22 transfer RNA genes, and a major non-coding control region 994 bp in length (D-loop region). The arrangement of these genes is the same as that found in the Siluriformes (Saitoh et al. 2003; Liu et al. 2019). All the protein initiation codons are ATG, except for cox1 that begins with GTG. The complete mitogenomes of *C. panda* and other 18 species from Siluriformes were used for phylogenetic analysis. The Neighbor-Joining tree builded by Geneious with Tamura–Nei (genetic distance model) and global aligment with free end gaps (aligment type) showed all species belong to 9 genera are divided into three groups (Siluridae, Loricariidae and Callichthyidae), and the *C. panda* was clustered with other species from genus *Corydoras* (Figure 1). *Corydoras panda* external morphological feature classification is consistent with the molecular classification results, so the information of the mitogenome could be used for future phylogenetic analysis and identification of *Corydoras* species (Betancur et al. 2017).

**Figure 1.**
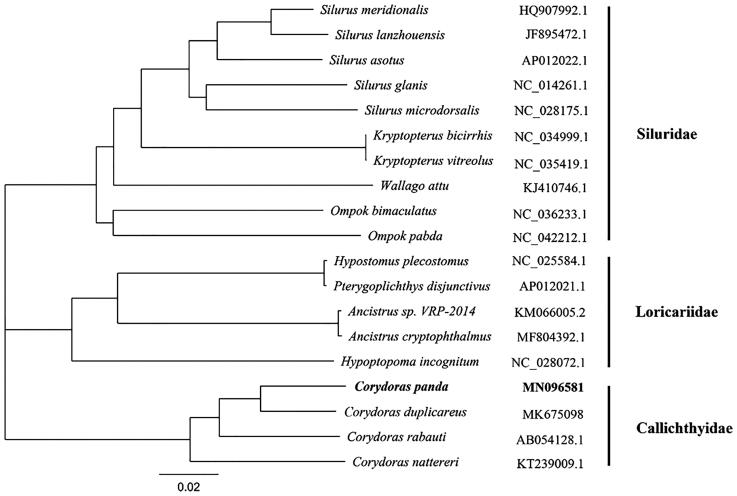
Neighbor-Joining phylogenetic tree based on the complete mitochondrial genome sequence. *Note:* the bold Latin name represents the species in this study. The codes followed the Latin names were GenBank accession numbers for each mitogenomes.
